# Long-term follow-up of patients with acute myeloid leukemia undergoing allogeneic hematopoietic stem cell transplantation after primary induction failure

**DOI:** 10.1038/s41408-023-00953-0

**Published:** 2023-12-10

**Authors:** Miriam Mozaffari Jovein, Gabriele Ihorst, Jesús Duque-Afonso, Ralph Wäsch, Hartmut Bertz, Claudia Wehr, Justus Duyster, Robert Zeiser, Jürgen Finke, Florian Scherer

**Affiliations:** 1 Department of Medicine I, Medical Center—University of Freiburg, Faculty of Medicine, University of Freiburg, Freiburg, Germany; 2 Clinical Trials Unit, Biometry and Statistics, University of Freiburg, Faculty of Medicine, University of Freiburg, Freiburg, Germany; 3 German Cancer Consortium (DKTK) partner site Freiburg and German Cancer Research Center (DKFZ), Heidelberg, Germany

**Keywords:** Stem-cell therapies, Disease-free survival, Acute myeloid leukaemia, Stem-cell research

## Abstract

Primary induction failure (PIF) in acute myeloid leukemia (AML) patients is associated with poor outcome, with allogeneic hematopoietic stem cell transplantation (HCT) being the sole curative therapeutic option. Here, we retrospectively evaluated long-term outcomes of 220 AML patients undergoing allogeneic HCT after PIF who never achieved remission, and identified clinical and molecular risk factors associated with treatment response and ultimate prognosis. In this high-risk population, disease-free survival was 25.2% after 5 years and 18.7% after 10 years, while overall survival rates were 29.8% and 21.6% after 5 and 10 years of HCT, respectively. 10-year non-relapse mortality was 32.5%, and 48.8% of patients showed disease relapse within 10 years after allogeneic HCT. Adverse molecular risk features determined at initial diagnosis, poor performance status at the time of allogeneic HCT, and long diagnosis-to-HCT intervals were associated with unfavorable prognosis. Collectively, our data suggests that immediate allogeneic HCT after PIF offers long-term survival and cure in a substantial subset of cases and that high-risk AML patients who never achieved complete response during induction might benefit from early donor search.

## Introduction

Allogeneic hematopoietic stem cell transplantation (HCT) is the standard consolidation treatment for most patients with adverse-risk acute myeloid leukemia (AML) [1, 2]. Outcomes of patients with AML undergoing allogeneic HCT have significantly improved over the last decades, mostly due to advances in stem cell harvesting modalities, supportive care, and infection management [3–5]. Furthermore, the introduction of reduced toxicity conditioning (RTC) regimens has made allogeneic HCT accessible for less fit and elderly patients [6–10]. Achieving complete remission (CR) before allogeneic HCT has been associated with improved clinical outcomes [11, 12]. Thus, AML patients with primary induction failure (PIF) who never achieve CR are often not considered for immediate allogeneic HCT and receive various other re-induction strategies, although allogeneic HCT represents the only curative option for these patients [2, 13–16]. Recently, Stelljes et al. reported that patients with relapsed/refractory AML had similar survival rates regardless of whether they proceeded directly to allogeneic HCT or underwent intensive remission induction prior to HCT within the ASAP trial [16]. Yet, median follow-up of this study was 37 months and the value of immediate allogeneic HCT in this clinical setting for induction of long-term response and durable remission is largely unclear.

In this single-center retrospective analysis, we explored long-term outcomes of 220 AML patients with PIF undergoing allogeneic HCT with active disease at our institution over a period of 30 years between 1989 and 2019, and investigated molecular and clinical features associated with clinical prognosis.

## Subjects and methods

### Patient selection and study design

We evaluated clinical and molecular data from 220 patients receiving allogeneic HCT after PIF at the University Medical Center Freiburg (Germany) between 1989 and 2019, with PIF being defined as never achieving CR during induction or re-induction, assessed either by cytomorphology or molecular measurable residual disease (MRD), as defined by the NCI (Version 23.06d, Code C70622) [2]. Patients provided written informed consent for the use of their data for clinical research after approval by the local Ethics Committee (22-1490-S1-retro). All analyses were performed in accordance with the Declaration of Helsinki.

Clinical and molecular examinations as well as laboratory analyses were performed as part of standard clinical care. Patients were treated at the discretion of the treating physician and according to institutional standards and national/international guidelines. Molecular risk at diagnosis and response was categorized according to the respective guidelines and criteria applicable at the time the patient was treated [17]. Throughout the manuscript, analyses were performed considering the entire cohort as well as distinct transplant periods: (i) 1989–2000, (ii) 2001–2010, and (iii) 2011–2019 (Table 1).

**Table 1:** Patient and disease specific characteristics.

Year of transplantation	1989–2019	1989–2000	2001–2010	2011–2019
Total *n* (%)	220 (100%)	36 (16.4%)	108 (49.1%)	76 (34.5%)
Age (years), median (range)	55 (20–75)	42 (25–64)	57 (22–75)	57 (20–74)
Female, *n* (%)	92 (41.8%)	14 (38.9%)	44 (40.7%)	34 (44.7%)
Molecular risk group				
Favorable, *n* (%)	2 (0.9%)	0 (0%)	0 (0%)	2 (2.6%)
Intermediate, *n* (%)	60 (27.3%)	10 (27.8%)	30 (27.8%)	21 (27.6%)
Adverse, *n* (%)	77 (35%)	8 (22.2%)	33 (30.6%)	36 (47.4%)
Missing molecular risk, *n* (%)	81 (36.8%)	18 (50%)	45 (41.7%)	17 (22.4%)
Blast count (BM) before conditioning therapy, median % (range)	36% (0–95%)	50% (8–90%)	40% (0–95%)	28% (0–90%)
Present, *n* (%)	184 (83.6%)	13 (63.9%)	94 (87%)	67 (88.2%)
Aplasia, *n* (%)	18 (8.2%)	1 (2.8%)	9 (8.3%)	8 (10.5%)
n.a., *n* (%)	18 (8.2%)	12 (33.3%)	5 (4.6%)	1 (1.3%)
Circulationg blast count (PB) before conditioning therapy, median % (range)	5% (0–98%)	5.5% (0–73%)	6% (0–98%)	2.5% (0–98%)
Present, *n* (%)	114 (51.8%)	9 (25%)	65 (60.2%)	40 (52.6%)
Absent, *n* (%)	91 (41.4%)	13 (36.1%)	42 (38.9%)	36 (47.4%)
n.a., *n* (%)	15 (6.8%)	14 (38.9%)	1 (0.9%)	0 (0%)
ECOG score				
0–1, *n* (%)	173 (78.6%)	9 (25%)	95 (88%)	69 (90.8%)
2–4, *n* (%)	22 (10%)	2 (5.6%)	13 (12%)	7 (9.2%)
n.a., *n* (%)	25 (11.4 %)	25 (69.4 %)	0 (0 %)	0 (0%)
HCT-CI, median (range)	3 (0–12)	n.a.	3 (0–7)	3 (0–12)
0–3, *n* (%)	56 (25.5%)	n.a.	21 (19.4%)	35 (46.1%)
≥4, *n* (%)	34 (15.5%)	n.a.	15 (13.9%)	19 (25%)
n.a., *n* (%)	130 (59.1%)	36 (100%)	72 (66.7%)	22 (28.9%)
AML type				
De novo AML, *n* (%)	135 (61.4%)	20 (55.6%)	63 (58.3%)	52 (68.4%)
sAML, *n* (%)	65 (29.5%)	12 (33.3%)	36 (33.3%)	17 (22.4%)
MDS, *n* (%)	55 (84.6 %)	12 (100%)	30 83.3%)	13 (76.5%)
MPN, *n* (%)	7 (10.8%)	0 (0%)	3 (8.3 %)	4 (23.5%)
MDS/MPN Overlap, *n* (%)	3 (4.6 %)	0 (0%)	3 (8.3%)	0 (0%)
t-AML, *n* (%)	20 (9.1%)	4 (11.1%)	9 (8.3%)	7 (9.2%)
Time from primary disease to tAML (years), median (range)	6.5 (0.5–20)	5.6 (0.5–11.2)	3 (1.4–18.9)	10.6 (2.0–20)
Number of treatment lines before HCT, median (range)	2 (1–6)	2 (1–6)	2 (1–5)	2 (1–5)
n.a., *n* (%)	14 (38.9%)	13 (36.1%)	1 (0.9%)	0 (0%)
Time from diagnosis to HCT (months), median (range)	3.48 (0.48–14.52)	3.96 (1.08–12.96)	3.72 (0.48–14.52)	2.88 (0.48–13.08)

Shown are patient and disease specific characteristics for the entire cohort (*n* = 220, column B) as well as for the three sub-cohorts according to the year of transplantation (column C: 1989–2000, *n* = 36; column D: 2001–2010, *n* = 108; column E: 2011–2019, *n* = 76).

*BM* bone marrow, *n.a.* not assessed, *PB* peripheral blood, *ECOG* Eastern Cooperative Oncology Group Score, *HCT-CI* Hematopoietic Cell Transplantation-specific Comorbidity Index, *AML* acute myeloid leukemia, *sAML* secondary acute myeloid leukemia, *MDS* myelodysplastic syndrome, *MPN* myeloproliferative neoplasm syndrome, *tAML* treatment-related acute myeloid leukemia, *HCT* hematopoietic cell transplantation.

### Statistical analysis

Data was collected prospectively and analyzed in a retrospective fashion. Primary endpoints were disease-free survival (DFS) and overall survival (OS). Other endpoints were time to relapse and time to non-relapse mortality (NRM). DFS was calculated from allogeneic HCT to AML relapse or death from any cause, OS was calculated from allogeneic HCT to death from any cause. NRM was calculated from allogeneic HCT to death without previous relapse. In case of a missing event, patients were censored at the last known follow-up time point. Time-to-event variables were visualized using the Kaplan–Meier method and log-rank tests were applied to evaluate survival differences. Curves for NRM and time to relapse were calculated using a cumulative-incidence model with the opposing event considered as competing. Univariate and multivariate risk analyses were conducted using Cox proportional hazards regression for OS and DFS.

All statistical analyses were performed using R-Studio 4.0.0 (2020-04-24) and Rx64 4.0.0 (2020-04-24). For log-rank and Cox regression analyses, the survival- package was used. For cumulative-incidence calculation, the cmprsk-package was used. *P* < 0.05 were considered statistically significant. Data analysis and graph generation was conducted using the dplyr and survminer-packages, packages of the R-tidyverse, and Prism Graph Pad 5.0.

## Results

We enrolled 220 AML patients in our study, undergoing allogeneic HCT after PIF between 1989 and 2019 (1989–2000: *n* = 36; 2001–2010: *n* = 108; 2011–2019: *n* = 76). Detailed patient, disease, and transplant-related characteristics are summarized in Tables 1 and 2. Median age of patients was 55 (range: 20–75), increasing from 42 in the first decade (1989–2000) to 57 in the second (2001–2010) and third decade (2011–2019, Table 1). This correlated with the proportion of patients treated with RTC, from 13.9% between 1989 and 2000 to 61.8% between 2011 and 2019 (Table 2). Molecular risk was assessed in 63.2% of patients, while molecular information was missing in 36.8% of AML cases. The proportion of patients with information on the molecular risk group increased over time with the availability of the respective technologies (Table 1) [17]. Most patients were diagnosed with de novo AML (61.4%), while 29.5% had secondary AML (sAML) emerging from myelodysplastic syndrome (MDS) or myeloproliferative neoplasm (MPN), and 9.1% of patients suffered from treatment-related AML (tAML). Patients received in median two lines of treatment before allogeneic HCT (range: 1–6), median time from diagnosis to allogeneic HCT was 3.48 months (range: 0.48–14.52 months) (Table 1). Unmodified myeloablative conditioning regiments (MAC) were used in 92 (41.8%) patients, while 125 (56.8%) patients were treated with RTC, and 3 (1.4%) patients received RIC regimens (Table 2). GvHD prophylaxis regimens consisted of either cyclosporin A (CsA) in combination with Alemtuzumab (35.9%) or anti-T lymphocyte globulin (ATG (Grafalon), 33.2%), or different strategies including mycophenolate-Mofetil (MMF) or methotrexate (30.9%) (Table 2). In 35% of cases, patients received stem cells from matched related donors (MRD), in 41.1% from matched unrelated donors (Table 2).

**Table 2:** Donor and transplant specific characteristics.

Year of transplantation	1989–2019	1989–2000	2001–2010	2011–2019
Total *n* (%)	220 (100%)	36 (16.4%)	108 (49.1%)	76 (34.5%)
Conditioning therapy*				
unmodified MAC, *n* (%)	92 (41.8%)	31 (86.1%)	35 (32.4%)	26 (34.2%)
RTC MAC, *n* (%)	125 (56.8%)	5 (13.9%)	73 (67.6%)	47 (61.8%)
RIC, *n* (%)	3 (1.4%)	0 (0%)	0 (0%)	3 (4.0%)
GvHD prophylaxys				
CsA + Alemtuzumab, *n* (%)	79 (35.9%)	0 (0%)	63 (58.3%)	16 (21.1%)
CsA + ATG, *n* (%)	73 (33.2%)	13 (36.1%)	24 (22.2%)	36 (47.4%)
Others**, *n* (%)	68 (30.9%)	23 (63.9%)	21 (19.4%)	24 (31.6%)
Incidence of GvHD				
aGvHD, *n* (%)	122 (55.5%)	17 (47.2%)	60 (55.6%)	45 (59.2%)
cGvHD, *n* (%)	66 (30%)	14 (38.9%)	34 (31.5%)	18 (23.7%)
cGvHD n.a., *n* (%)	52 (23.6%)	15 (41.7%)	18 (16.7%)	19 (25.0%)
Immunosuppression free survival (years), median (range)	0.2 (0–20.9)	0.1 (0–20.9)	0.7 (0–17.2)	0.1 (0–7.1)
Donor match				
MRD, *n* (%)	77 (35%)	19 (52.8%)	34 (31.5%)	24 (31.6%)
MUD, *n* (%)	97 (44.1%)	12 (33.3%)	49 (45.4%)	36 (47.4%)
MMRD, *n* (%)	2 (1%)	1 (2.8%)	1 (0.9%)	0 (0%)
MMUD, *n* (%)	44 (20%)	4 (11.1%)	24 (22.2%)	16 (21.1%)
Donor sex				
Female, *n* (%)	100 (45.5%)	17 (47.2%)	47 (43.5%)	36 (47.4%)
Donor recipient sex match				
Matched, *n* (%)	128 (58.2%)	23 (63.9%)	67 (62%)	38 (50%)
Male recipient, female donor, *n* (%)	50 (22.7%)	8 (22.2%)	22 (20.4%)	20 (26.3%)
Female recipient, male donor, *n* (%)	42 (19.1%)	5 (13.9%)	19 (17.6%)	18 (23.7%)
Donor age (years), median (range)	38 (14–74)	36 (21–59)	40 (14–74)	34 (19–69)
Unknown, *n* (%)	12 (5.5%)	10 (27.8%)	2 (1.9%)	0 (0%)
≤ 25 years, *n* (%)	30 (13.6%)	3 (8.3%)	12 (11.1%)	15 (19.7%)
26–50 years, *n* (%)	143 (65%)	20 (55.6%)	74 (68.5%)	49 (64.5%)
≥ 51 years, *n* (%)	35 (15.9%)	3 (8.3%)	20 (18.5%)	12 (15.8%)
CMV risk constellation				
Positive recipient, negative donor, *n* (%)	52 (23.6%)	9 (25%)	30 (27.8%)	13 (17.1%)
Negative recipient, positive donor, *n* (%)	22 (10%)	2 (5.6 %)	11 (10.2 %)	9 (11.8%)
Both positive, *n* (%)	82 (37.3%)	6 (16.7%)	46 (42.6%)	30 (39.5%)
Both negative, *n* (%)	62 (28.2%)	17 (47.2%)	21 (19.4%)	24 (31.6%)
Unknown, *n* (%)	2 (0.9%)	2 (5.6%)	0 (0%)	0 (0%)
Graft source				
BM, *n* (%)	18 (8.2%)	15 (41.7%)	2 (1.9%)	1 (1.3%)
PBSC, *n* (%)	202 (91.8%)	21 (58.3%)	106 (98.1%)	75 (98.7%)
Median CD34+ count				
CD34+ cells*10^6/kg bw (range)	6.6 (0.7–34)	5.3 (2.5–14)	6.6 (1.1–34)	6.7 (0.7–17)
Unknown graft size, *n* (%)	18 (8.2%)	18 (50%)	0 (0%)	0 (0%)

Shown in this table are donor and transplant specific characteristics for the entire cohort (*n* = 220, column B) as well as for the three sub-cohorts according to the year of transplantation (column C: 1989–2000, *n* = 36; column D: 2001–2010, *n* = 108; column E: 2011–2019, *n* = 76).

*MAC* myeloablative conditioning, *RTC* reduced toxicity myeloablative conditioning, *RIC* reduced intensity conditioning, *GvHD* graft-versus-host disease, *aGvHD* acute GvHD, *cGvHD* chronic GvHD, *CsA* Cyclosporin A, *ATG* anti-T lymphocyte globulin (Grafalon), *MRD* matched related donor, *MUD* matched unrelated donor, *MMRD* mismatched related donor, *MMUD* mismatched unrelated donor, *CMV* cytomegalovirus, *BM* bone marrow, *PBSC* peripheral blood stem cell, *MMF* Mycophenolate-Mofetil, *MTX* methotrexate.

*unmodified MAC protocols consisted of BU/CY, FLU/BU (4 days), TBI/Cy, TBI/VP16/CY, TT/BU/FLU (MAC); RTC MAC protocols consisted of FLU, FLU/BCNU/MEL, FLU/BCNU/TT, FLU/TT/MEL, TT/BU/FLU (modified), TT/FLU/Treo (MAC); RIC protocols consisted of TT/BU/FLU (RIC), FLU/TT, TT/FLU/Treo (RIC).

**others = neither ATG nor Alemtuzumab. Included are combinations of MMF and MTX.

**Fig. 1:**
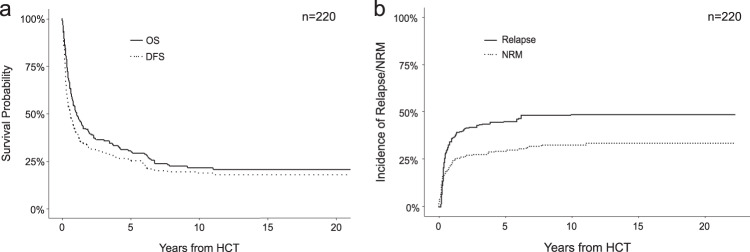
Clinical outcomes of the whole cohort. **a** Kaplan–Meier analysis of overall survival (continuous line) and disease-free survival (dotted line) of the entire patient cohort. **b** cumulative-incidence model of relapse rates (continuous line) and non-relapse mortality (dotted line) of the entire patient cohort. OS overall survival, DFS disease-free survival, NRM non-relapse mortality, HCT hematopoietic cell transplantation.

The median follow-up of our study was 8.5 years (range: 0.06–25.4 years). Disease-free survival at 1 year was reached in 39.8% of AML patients, in 25.2% after 5 and 18.7% after 10 years of allogeneic HCT (Fig. 1a and Table 3). Median OS was 1.05 years, while 1-year and 5-year OS were 50.4% and 29.8%, respectively, and 21.6% of patients were alive 10 years after allogeneic HCT (Fig. 1a and Table 3). Survival rates were largely stable over time. For example, 5-year OS in patients treated between 1998-2000 was 28.7%, in those receiving HCT between 2001 and 2010 28.7%, and 30.3% in patients treated between 2011 and 2019 (Table 3). Non-relapse mortality (NRM) for the whole cohort after 5 years was 29.3% and 32.5% after 10 years (Fig. 1b and Table 3). Acute graft versus host disease (aGvHD) was observed in 55.5% of patients, while 30% of patients suffered from chronic graft versus host disease (cGvHD) (Table 2). Relapse rates of the entire cohort after 5 and 10 years were 44.9% and 48.8%, respectively (Fig. 1b and Table 3). Over time, relapse rates varied slightly, with a 5-year relapse rate of 45.7% between 1998 and 2000, 50.9% in 2001–2010, and 33.3% between 2011 and 2019 (Table 3), reflecting small variations of risk factors within the patient populations.

**Table 3:** Overview of survival rates.

Year of transplantation	1989–2019	1989–2000	2001–2010	2011–2019
	**1 year**			
OS, *n*%	50.4%	40.2%	55.6%	46.2%
DFS, *n*%	39.8%	40.2%	41.7%	38.1%
NRM, *n*%	24.1%	25.7%	18.5%	31.7%
Relapse, *n*%	35.7%	34.3%	39.8%	30.3%
	**5 years**			
OS, *n*%	29.8%	28.7%	28.7%	30.3%
DFS, *n*%	25.2%	23.0%	25.9%	29.6%
NRM, *n*%	29.3%	31.4%	23.1%	37.1%
Relapse, *n*%	44.9%	45.7%	50.9%	33.3%
	**10 years**			
OS, *n*%	21.6%	25.8%	20.3%	n.a.
DFS, *n*%	18.7%	17.2%	18.5%	n.a.
NRM, *n*%	32.5%	34.3%	25.9%	n.a.
Relapse, *n*%	48.8%	48.6%	55.6%	n.a.

Highlighted in this table are overall survival, disease-free survival, non-relapse mortality and relapse rates in the whole cohort and sub-cohorts after 1, 5 and 10 years, respectively.

*OS* overall survival, *DFS* disease-free survival, *NRM* non-relapse mortality, *n.a.* not assessed.

At 1 month after HCT, 206 patients (93.6%) were alive and 59 patients (26.8%) were alive at the time of the final study analysis. The main cause of death after HCT was treatment failure and AML progression (57.8%), while infections (19.3%), GvHD (11.2%), and treatment-related toxicity (8.4%) were leading causes of NRM (Table 4). Secondary malignancies led to death in 1.9% of patients (Table 4).

**Table 4:** Causes of death.

Year of transplantation	1989–2019	1989–2000	2001–2010	2011–2019
Deceased patients, *n* (%)	161 (73.2%)	26 (72.2%)	87 (80.6%)	48 (63.2%)
Relapse/primary disease, *n* (%)	93 (57.8%)	15 (57.7%)	56 (64.4%)	21 (43.8%)
Non-relapse mortality, *n* (%)				
Infections, *n* (%)	31 (19.3%)	6 (23.1%)	18 (20.7%)	11 (22.9%)
Organ failiure/Toxicity, *n* (%)	11 (8.4%)	3 (11.5%)	4 (4.6%)	8 (16.7%)
GvHD total, *n* (%)	18 (11.2%)	1 (3.8%)	5 (5.7%)	5 (10.4%)
aGvHD, *n* (%)	8 (5%)	0 (0%)	3 (3.4%)	3 (6.3%)
cGvHD, *n* (%)	10 (6.2%)	1 (3.8%)	2 (2.3%)	2 (4.2%)
Secondary malignancy, *n* (%)	3 (1.9%)	1 (3.8%)	2 (2.3%)	0 (0%)
Graft failure, *n* (%)	2 (1.2%)	0 (0%)	1 (1.2%)	1 (2.1%)
Unknown, *n* (%)	3 (1.9%)	0 (0%)	1 (1.2 %)	2 (4.2%)

Shown are the causes of death in 161 patients from the entire cohort and the sub-cohorts. Causes of death include primary disease, infections, organ failure or toxicity, GvHD, graft failure, secondary malignancies and unknown causes.

*GvHD* graft-versus-host disease, *aGvHD* acute graft-versus-host disease, *cGvHD* chronic graft-versus-host disease.

**Fig. 2:**
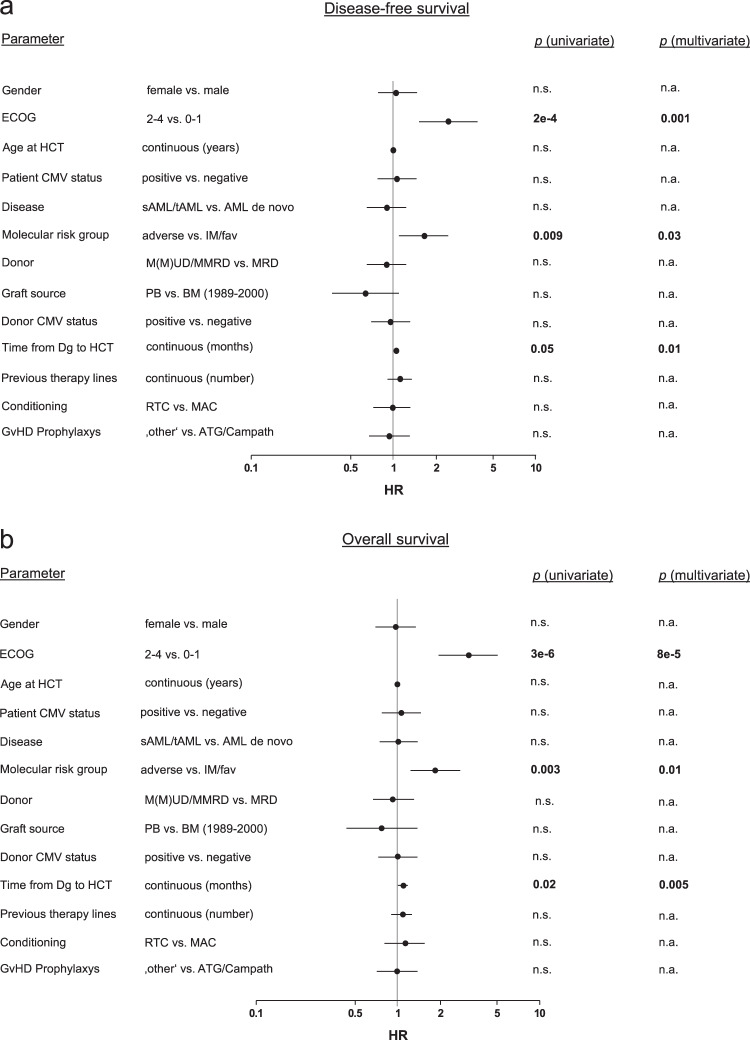
Univariate and multivariate survival analysis. Forest plots showing hazard ratios for DFS (**a**) and OS (**b**) estimated by univariate and multivariate Cox proportional hazards regression outcome analyses, incorporating patient-, disease-, and treatment-specific characteristics. *P*-values were calculated using the Walden test. ECOG Eastern Cooperative Oncology Group Score, HCT hematopoietic cell transplantation, CMV cytomegalovirus, AML acute myeloid leukemia, sAML secondary acute myeloid leukemia, IM intermediate, fav favorable, n.a. not assessed, M(M)UD (mis)matched unrelated donor, MMRD mismatched related donor, MRD matched related donor, PB peripheral blood, BM bone marrow, Dg diagnosis, RTC reduced toxicity conditioning, MAC myeloablative conditioning, ATG anti-T lymphocyte globulin, Campath, Alemtuzumab.

**Fig. 3:**
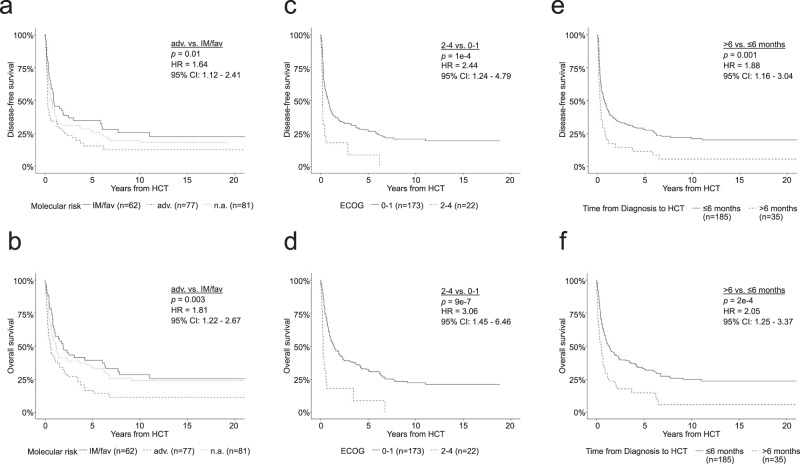
Association of risk factors with survival. Kaplan–Meier analyses of DFS (**a**) and OS (**b**) in patients with adverse molecular risk (*n* = 77) vs. intermediate/favorable molecular risk (*n* = 62). Patients with unknown molecular risk are also shown (*n* = 81). Kaplan–Meier analyses of DFS (**c**) and OS (**d**) in patients with ECOG 0–1 (*n* = 173) vs. 2–4 (*n* = 22). Kaplan–Meier analyses of DFS (**e**) and OS (**f**) in patients with Diagnosis-to-HCT interval of less than 6 months (*n* = 185) vs. at least 6 months (*n* = 35). OS overall survival, DFS disease-free survival, HCT hematopoietic cell transplantation, ECOG Eastern Cooperation Oncology Group score, IM intermediate, fav favorable, n.a. not assessed.

In Cox regression analyses incorporating known molecular and clinical risk factors, poor performance status (ECOG > 1; DFS: *p* = 0.001, HR: 2.3, 95% CI: 1.4–3.7; OS: *p* = 8*10^−5^, HR: 2.8, 95% CI: 1.7–4.6), adverse molecular risk at AML diagnosis (vs. intermediate/favorable risk; DFS: *p* = 0.03, HR: 1.6, 95% CI: 1.1–2.4; OS: *p* = 0.01, HR: 1.7, 95% CI: 1.1–2.6), and long diagnosis-to-HCT interval (DFS: 0.01, HR: 1.1, 95% CI: 1.0–1.2; OS: *p* = 0.005, HR: 1.1, 95% CI: 1.0–1.2) were strongly and independently associated with unfavorable DFS and OS (Fig. 2a, b). This was confirmed by log rank analyses, revealing that DFS and OS was shorter in patients with adverse molecular risk factors (vs. intermediate/favorable risk; DFS: *p* = 0.01, HR: 1.64, 95% CI: 1.12–2.41; OS: *p* = 0.003, HR: 1.81, 95% CI: 1.22–2.67, Fig. 3a, b), ECOG > 1 (DFS: *p* = 1*10^−4^, HR: 2.44, 95% CI: 1.24–4.79; OS: *p* = 9*10^−7^, HR: 3.06, 95% CI: 1.45–6.46, Fig. 3c, d), and a time from diagnosis to HCT greater than 180 days (DFS: *p* = 0.001, HR: 1.88, 95% CI: 1.16–3.04; OS: *p* = 2*10^−4^, HR: 2.05, 95% CI: 1.25–3.37, Fig. 3e, f). Importantly, the association between these clinical features and outcomes was maintained when restricting the analyses to patients proceeding to allogeneic HCT after PIF during first line therapy (Supplementary Table 1 and Supplementary Fig. 1). Notably, age, donor type (MRD vs. MMUD/MUD/MMRD), GvHD-prophylaxis, conditioning regimen (RTC vs. MAC), and AML subtypes did not influence clinical outcomes (Fig. 2). The presence of circulating blasts before conditioning has previously been shown to be associated with unfavorable outcomes [18]. Our data support this finding, we also found shorter DFS and OS in patients with circulating blasts compared to patients with no measurable blasts in peripheral blood (Log-rank analyses: DFS: *p* = 2*10^−5^, HR: 2.00, CI: 1.44–2.7; OS: *p* = 0.001, HR: 1.72, CI: 1.25–2.37, Supplementary Fig. 2).

## Discussion

Primary induction failure is associated with poor prognosis in patients with AML [17, 19–22]. In this situation, re-induction with cytarabine- or venetoclax-based therapies followed by allogeneic HCT in CR is considered the standard procedure to induce long-term remissions [23]. However, Stelljes et al. recently demonstrated in a large randomized phase III trial (ASAP trial), which enrolled 281 patients with relapsed/refractory AML, that re-induction with high-dose cytarabine and mitoxantrone before allogeneic HCT did not result in higher early overall response rates or a survival advantage compared to immediate conditioning and allogeneic HCT without prior remission induction [16]. While the median follow-up of this study was relatively short with 37 months, we here add evidence that allogeneic HCT with active disease at time of transplantation can also lead to long-term remission and cure in a substantial subset of cases. In our retrospective single-center study, we analyzed 220 AML patients with PIF undergoing allogeneic HCT while not being in CR over a period of 30 years. Median OS for the whole group was 1.05 years, with a 5-year OS of 29.8%. These results are largely concordant with previous studies. For example, Craddock et al. demonstrated a 5-year OS of 22% in refractory AML patients who underwent unrelated donor transplantation between 1994 and 2006 [8]. In a retrospective study led by Nagler et al. that included more than 3400 patients with relapsed/refractory AML treated with allogeneic HCT from unrelated donors, 2-year DFS and OS were 29.8% and 36.5% after a median follow-up of 4 years [24]. Importantly, our results were also comparable to studies investigating survival in high-risk AML patients undergoing HCT in CR/CRi [25] and might be superior to those investigating treatment with hypomethylating agents and venetoclax without allogeneic HCT in relapsed/refractory AML patients (Median OS: 5 months, 60-day mortality rate: 33.3%) [26]. Collectively, our data indicates that immediate allogeneic HCT in refractory AML patients with active disease might be a reasonable alternative to repeated remission induction strategies before proceeding to HCT, and represents a feasible strategy to induce long-term survival and durable remissions.

The other major finding of our study is the association of outcomes with adverse molecular risk factors at AML diagnosis, performance status, and the diagnose-to-HCT interval, while age and donor type did not correlate with prognosis in our cohort. The role of most of these risk factors for outcome prediction after allogeneic HCT is discussed controversially in previous studies. For example, the prognostic value of donor types has been addressed by several register trials over the last years, showing either no significant difference between siblings and unrelated donors [27, 28], more favorable outcomes in patients undergoing HCT with unrelated donors [29], or worse prognosis with haploidentical donors [29, 30]. In our analysis, we found no significant association of donor types with patient outcomes, likely due to advances in treatment and transplant modalities over decades that might contribute to omitting the influence of donor source. Similarly, in opposite to other studies in AML and MDS, patient’s age was not prognostic in our analysis [31, 32]. On the other hand, the lack of an association between conditioning protocols (RTC vs. MAC) and outcomes after HCT, the predictive value of molecular risk factors and the performance status have been demonstrated before and are in line with our results [9, 14, 33–35]. Importantly, we found that patients with a long diagnosis-to-HCT interval show shorter DFS and OS, indicating that a delay of allogeneic HCT, repetitive re-induction, and prolonged presence of the disease might contribute to impaired patient conditions before HCT [8, 27]. Separately, higher disease activity before conditioning, measured by the presence of circulating blasts, led to unfavorable outcomes, which is in line with the findings reported by Duval et al. [18].

Our study harbors several limitations, which include its retrospective nature, heterogeneity of the patient cohort, in particular a younger median age in patients treated between 1989 and 2000, and the lack of various clinical and molecular parameters in a substantial subset of patients. For example, information on molecular risk or the HCT-CI score is largely missing in patients treated within the first decade of the study. Further limitations include heterogenic therapeutic approaches such as an increased use of RTC in the last decade, the unavailability of CsA/Alemtuzumab between 1989–2000, and variable graft sources over time (41.7% bone marrow graft between 1989–2000 and only 1.3% between 2011–2019). However, to our knowledge, this study offers the longest follow up of AML patients with PIF undergoing allogeneic HCT while not being in CR.

Collectively, we demonstrated that immediate allogeneic HCT in AML patients with active disease represents a valid alternative to intensive remission induction and provides long-term survival and cure in a significant proportion of patients, highlighting the importance of allogeneic HCT as the most effective treatment option in this high-risk group. Our data further suggests starting donor search at AML diagnosis and to immediately proceed with conditioning and allogeneic HCT in refractory patients whenever a donor is available.

### Supplementary information

Supplemental material

## Data Availability

The analyzed patient, disease and treatment characteristics are presented in Tables 1–4. More granular, anonymized data is available from the authors upon request.

## Code availability

R-code can be obtained from the authors upon request.
