# Supplementary material for: Long-term follow-up of patients with acute myeloid leukemia undergoing allogeneic hematopoietic stem cell transplantation after primary induction failure
Source: Blood Cancer J. 2023 Dec 10;13(1):179. doi: 10.1038/s41408-023-00953-0 (PMC10710471; doi:10.1038/s41408-023-00953-0)
Supplement: Supplementary file 1 — Supplemental material [file 41408_2023_953_MOESM1_ESM.pdf]

Supplementary Material for

Long-term follow-up of patients with acute myeloid leukemia undergoing allogeneic hematopoietic stem cell transplantation after primary induction failure

Miriam Mozaffari Jovein, Gabriele Ihorst, Jesús Duque-Afonso, Ralph Wäsch, Hartmut Bertz, Claudia Wehr, Justus Duyster, Robert Zeiser, Jürgen Finke, Florian Scherer ^

^ Corresponding author. Email: florian.scherer@uniklinik-freiburg.de

Supplementary Tables

a. DFS					
**Risk factor**	**Values**	**Hazard Ratio**	**lower 95% CI**	**upper 95% CI**	**p (univariate)**
ECOG	2-4 vs. 0-1	8.65	2.77	27.03	**0.0002**
Molecular risk	adverse vs. IM/fav	2.31	1.13	4.73	**0.02**
Diagnosis-to-HCT interval	continuous (months)	1.18	1.04	1.33	**0.008**
Diagnosis-to-HCT interval	≤6 vs >6 months	2.1	1.06	4.19	**0.03**
**b. OS**					
**Risk factor**	**Values**	**Hazard Ratio**	**lower 95% CI**	**upper 95% CI**	**p (univariate)**
ECOG	2-4 vs. 0-1	11.27	3.33	38.17	**0.0001**
Molecular risk	adverse vs. IM/fav	2.41	1.18	4.92	**0.02**
Diagnosis-to-HCT interval	continuous (months)	1.23	1.08	1.39	**0.001**
Diagnosis-to-HCT interval	≤6 vs >6 months	2.31	1.16	4.6	**0.02**
DFS disease-free survival, *ECOG* Eastern Cooperation Oncology Group, *CI* Confidence Interval, *IM* intermediate, *fav* favorable, *vs.* versus, *n.a.* not assessed, *Dg* diagnosis, *HCT* hematopoietic cell transplantation, *OS* overall survival.					

Supplementary Table 1. Cox regression analysis in patients with one previous therapy line. (a,b) Results of Cox proportional hazards regression for disease-free survival **(a)** and overall survival **(b)** in AML patients undergoing allogeneic HCT after the first induction line. Listed are univariate *p*-values, hazard ratios, and confidence intervals for the factors ECOG 0-1 vs. 2-4, molecular risk (adverse vs. IM/fav), and diagnosis-to-HCT interval (continuous and ≤6 vs. >6 months). *p*-values were estimated by Walden test.

Supplementary Figures

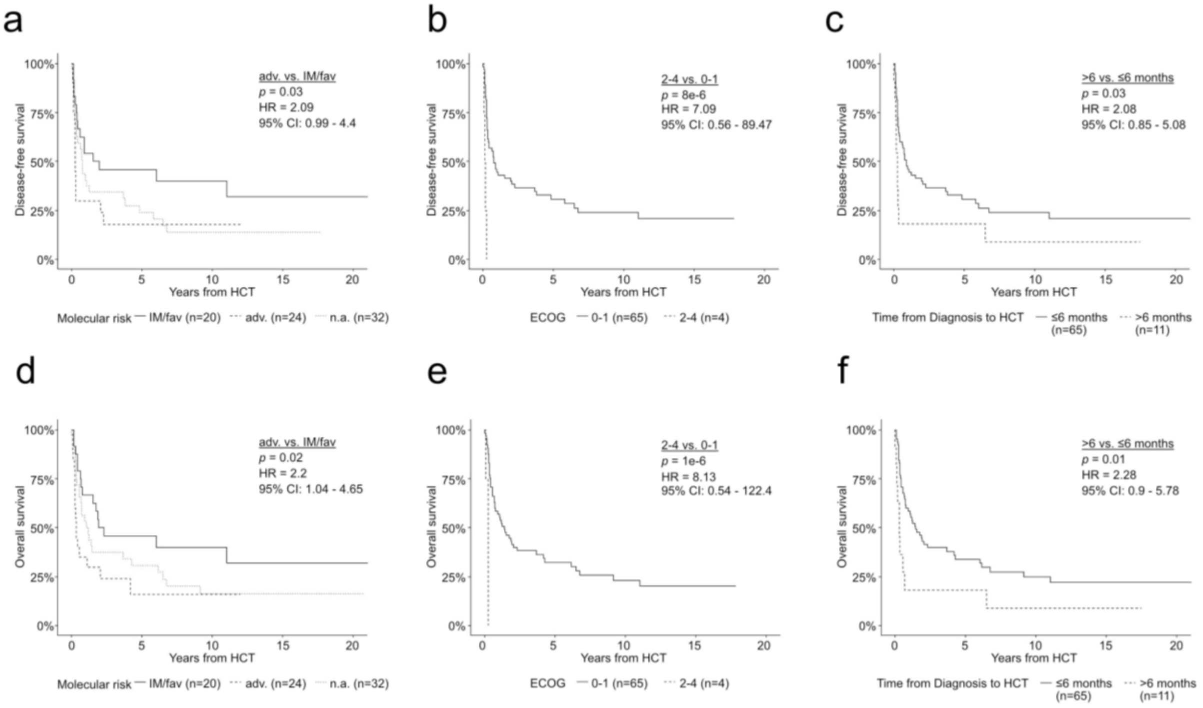

Supplementary Figure 1. (a-c) Kaplan-Meier analyses of disease-free survival in a subset of *n*=76 patients who received only one induction therapy line before undergoing HCT, stratified by **(a)** molecular risk profile with intermediate/favorable (*n*=20), adverse (*n*=24), and unknown (n.a.) risk (*n*=32), **(b)** ECOG with a score of ≤ 1 (*n*=65) vs. 2-4 (*n*=4), and **(c)** diagnosis-to-HCT interval comparing up to 6 months (*n*=65) to over 6 months (*n*=11). **(d-f)** Kaplan-Meier analyses of overall survival in a subset of *n*=76 patients who received only one induction therapy line before undergoing HCT, stratified by **(d)** molecular risk profile with intermediate/favorable (*n*=20), adverse (*n*=24), and unknown (n.a.) risk (*n*=32), **(e)** ECOG with a score of ≤ 1 (*n*=65) vs. 2-4 (*n*=4), and **(f)** diagnosis-to-HCT interval comparing up to 6 months (*n*=65) to over 6 months (*n*=11). *OS* overall survival, *DFS* disease-free survival, *HCT* hematopoietic cell transplantation, *ECOG* Eastern Cooperation Oncology Group score, *IM* intermediate, *fav* favorable, *n.a.* not assessed.

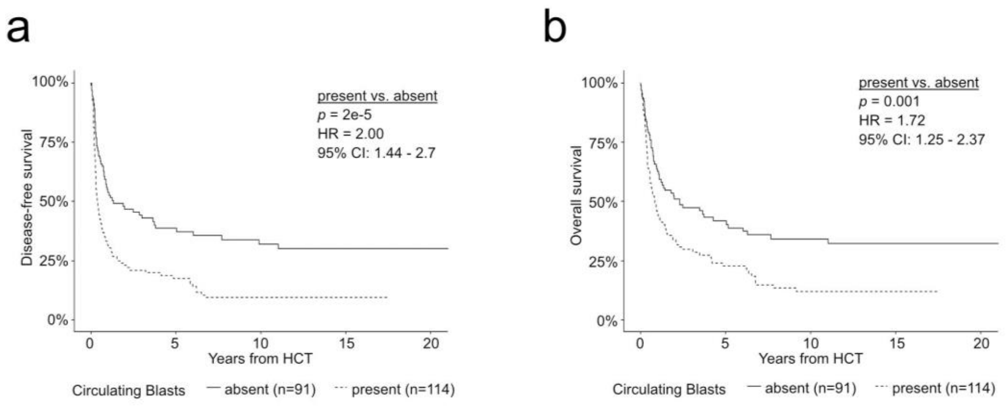

Supplementary Figure 2. (a) Kaplan-Meier analysis of disease-free survival in the presence (*n*=44) vs. in the absence (*n*=114) of circulating blasts prior to conditioning therapy and allogeneic HCT. **(b)** Kaplan-Meier analysis of overall survival in the presence (*n*=44) vs. in the absence (*n*=114) of circulating blasts prior to conditioning therapy and allogeneic HCT. *OS* overall survival, *DFS* disease-free survival, *HCT* hematopoietic cell transplantation.
